# A53 FECAL BIOACTIVE PHOSPHOLIPIDS AS TRIGGERS OF INTESTINAL HYPERSENSITIVITY IN IRRITABLE BOWEL SYNDROME

**DOI:** 10.1093/jcag/gwac036.053

**Published:** 2023-03-07

**Authors:** J Pujo, G De Palma, J Lu, G Rueda, S Collins, P Bercik

**Affiliations:** Medicine, McMaster, Hamilton, Canada

## Abstract

**Background:**

Chronic abdominal pain is the most common complaint of patients with gastrointestinal disorders. Its treatment is of limited efficacy as the pathophysiology is poorly understood. The gut microbiome has been shown to affect host physiology, including the neural system function, and growing evidence suggests that it plays an important role in pain perception. Gut microbiota produces a large variety of molecules that can regulate pain perception, such as histamine or bioactive lipids. Phospholipid mediators (PLM) are signaling molecules linked to neurogenic pain as by-products of inflammatory processes in mammals. But it is unknown whether bacteria can produce PLM and whether they play any role in visceral hyperalgesia.

**Purpose:**

To investigate whether PLM are present in stool of patients with chronic abdominal pain, and whether they have potential to induce visceral hypersensitivity.

**Method:**

Stool samples from 27 patients with irritable bowel syndrome (IBS) were collected both during periods of severe and minimal/no pain. The fecal concentration of two PLM (lipid A and lipid B) was determined by ELISA. The passage of lipid A and lipid B through the intestinal barrier was determined *ex vivo* using Ussing chambers and *in vivo* by their intracolonic instillation, and their levels assessed in the serosal compartment and the serum, respectively, by ELISA. We used primary cultures of sensory neurons from the dorsal root ganglia (DRG) of conventional mice (SPF) to study the neuronal activation *in vitro*. Calcium mobilization in DRG sensory neurons was measured by an inverted fluorescence microscope using a fluorescent probe Fluo-4 (1mM) after stimulation with different concentration of lipid A or lipid B. Visceral sensitivity *in vivo* after intracolonic instillation of combined lipids A and B was evaluated by colorectal distension.

**Result(s):**

The concentration of lipid A (p=0.001) and lipid B (p=0.002) in stool was significantly higher in IBS patients when experiencing severe abdominal pain compared to periods of minimal pain. The concentration of lipid A (p=0.03; p=0.07) and lipid B (p=0.018; p=0.018) in the serosal compartment and in the serum, respectively, was higher compared to the control condition. The percentage of neurons responding to lipid A or lipid B was significantly higher compared to the control condition, at all tested concentrations. Finally, combined lipid A and lipid B induced visceral hypersensitivity within 10 minutes (p=0.0433) and 90 minutes (p=0.0020) after intracolonic instillation.

**Conclusion(s):**

Our data suggest that PLM can be found in stool of patients with abdominal pain, that they cross the colonic barrier and increase visceral sensitivity. Further studies are needed to ascertain whether gut bacteria produce these PLM and investigate the precise mechanisms by which PLM induce hyperalgesia.

**Please acknowledge all funding agencies by checking the applicable boxes below:**

CIHR, Other

**Please indicate your source of funding;:**

Weston Family Foundation

**Disclosure of Interest:**

None Declared

